# Oxidative Damage and Mutagenic Potency of Fast Neutron and UV-B Radiation in Pollen Mother Cells and Seed Yield of *Vicia faba* L.

**DOI:** 10.1155/2013/824656

**Published:** 2013-08-28

**Authors:** Ekram Abdel Haliem, Hanan Abdullah, Asma A. AL-Huqail

**Affiliations:** ^1^Plant Cytogenetic and Molecular Biology, Botany Department, Science College, Zagazig University, P.O. Box 44519, Zagazig 308213, Egypt; ^2^Plant Physiology, Botany and Microbiology Department, Science College, King Saud University, P.O. Box 22452, Riyadh 11495, Saudi Arabia; ^3^Plant Physiology, Botany Department, Science College, Zagazig University, P.O. Box 44519, Zagazig 308213, Egypt

## Abstract

In recent years, there has been a great deal of attention toward free radicals, reactive oxygen species (ROS) generated by exposure of crop plant cells to physical radiations. Henceforth, the current study was planned to compare oxidative stress and mutagenic potential of different irradiation doses of fast neutron (FN) and UV-B on meiotic-pollen mother cells (PMCs), pollen grains (PGs) and seeds yielded from irradiated faba beans seedlings. On the cytogenetic level, each irradiation type had special interference with DNA of PMC and exhibited wide range of mutagenic action on the frequency and type of chromosomal anomalies, fertility of PGs and seed yield productivity based on the irradiation exposure dose and radiation sensitivity of faba bean plants compared with un-irradiated ones. On the molecular level, SDS-PAGE and RPAD-PCR analyses of seeds yielded from irradiated seedlings exhibited distinctive polymorphisms based on size, intensity, appearance, and disappearance of polypeptides bands compared with un-irradiated ones. The total values of protein and DNA polymorphisms reached 88% and 90.80% respectively. The neutron fluency (2.3 × 10^6^ n/cm^2^) and UV-B dose for 1 hr were recorded as bio-positive effects. The present study proved that genetic variations revealed by cytogenetic test could be supported by gene expression (alterations in RAPD and protein profiles).

## 1. Introduction

It has been known for many years that exposure of crop plant cells under natural conditions of growth and development to physical radiations such as ionizing FN and nonionizing UV-B resulted in excessive production of free radicals ROS [1, 2], respectively. These radiolytic ROS include a wide range of oxygen-radicals, such as superoxide anion (O_2_^∙−^), hydroxyl radical (^∙^OH), perhydroxyl radical (HO_2_^∙^), and hydrogen peroxide (H_2_O_2_) [3]. They are highly reactive due to the presence of unpaired valence shell electrons [4] and can result in noncontrolled oxidation in cells, cellular macromolecules compartments including DNA, proteins, lipids, and enzymes [5]. On the other hand, ROS-induced genotoxic damage can induce structural changes in DNA, such as chromosomal rearrangement, strand breaks, base deletions, pryrimidine dimers, cross-links and base modifications, mutations, and other genotoxic effects [5, 6]. 

Despite the ROS destructive activity, their production in plant tissues is controlled by the very efficient enzymatic and nonenzymatic antioxidant defense systems which serve to keep down the levels of free radicals, permitting them to perform useful biological functions without too much damage and act as a cooperative network employing a series of redox reactions [5, 7]. From these plants, leguminous especially faba bean plant which proved that it has high antioxidant activity due to that they contained phenolic and flavonoid compounds [8–10]. On the other hand, it has a diploid (2*n* = 12) and relatively large chromosomes. Therefore, it is important model system among the plant bioassays for monitoring or testing environmental pollutants as reviewed by the US Environmental Protection Agency (EPA) Gene Tox program [11] and can detect a wide range of genetic damage, including gene mutations, chromosome aberrations, and DNA strand breaks [12]. 

Biologically, FN differs from UV-B radiation in the way in which energy is distributed in irradiated tissues and their biological effects in the living cell [1]. Each type of these radiations can induce ROS in cell by special interference with cellular macromolecules (DNA and protein). The effects of these radiations vary depending on the applied dose and sensitivity of living plant cell to the action of radiation type [13]. The biological irradiation by FN based on the interaction with atoms or molecules in living cell, particularly water, to produce free radicals, which induce DNA deletions in nucleus and chromosome that range in size from a few base pairs to several megabases [14]. It is a potent DNA-damaging agent and more efficient in inducing biochemical modification of bases and double strand breaks in DNA by directly ionizing DNA itself or by indirect processes in which DNA reacts with numerous radiolytic reactive products that are generated in aqueous fluid surrounding DNA causing DNA base oxidation and DNA breaks formation (i.e., single-strand breaks, SSBs and double-strand breaks, DSBs) [13, 14]. All these modifications lead to protein denaturation which causes a conformational change in the structure and render them inactive [1]. On the other hand, the strong absorption of the UV-B at (280–320 nm) by DNA and protein in plant cells [15] based on photons which have enough energy to destroy chemical bonds between these macromolecules, causing a photochemical reaction which lead to generation of highly toxic reactive oxygen species (ROS) in cells [2]. Radiolytic ROS induce oxidative DNA damage by oxidative cross linking between adjacent pyrimidine bases forming cyclobutane-pyrimidine dimers (CPDs), 6-4 photoproducts (6-4PPs) and their Dewar valence isomers, that ultimately block the movement of DNA polymerases on DNA template [16–18] and also induce oxidative protein cross-links by alteration of the expression of several genes through nonspecific signaling pathways leading to protein destruction which often causes heritable mutations affecting various physiological processes [5].

It is important for detection of oxidative stress and mutagenic potential of various types of radiations on crop plants, to understand their biological consequences and their molecular action on chromosome, protein, and DNA of plant cell by introducing cytogenetic and molecular assays. Cytogenetic tests are considered to be indicator of cytotoxicity, genotoxicity, genetic variability, and estimation of the mutagen potency in meiotic PMCs and PGs [12, 19]. On the other hand, proteins being primary gene products of plant's DNA hence, any observed variation in protein systems induced by oxidative stresses or any mutagen is considered as a mirror for genetic variations [20]. Determination of protein MWs via polyacrylamide gel electrophoresis in the presence of sodium dodecyl sulfate (SDS-PAGE) is a universally used method in biomedical research [21]. Some studies used SDS-PAGE for detection of alterations in protein profiles occurring during irradiation by UV-B [15, 22]. Furthermore, DNA alterations based on random amplification of polymorphic DNA (RAPD) profiles are a useful biomarker assay for the evaluation of genotoxic and mutagenic effects of radiations on plants especially when the nucleotide sequence is not known [23]. The advantage of RAPD relies on its simplicity, rapidity, a small quantity of DNA, and its ability to generate more number of polymorphic bands, numerous polymorphisms [24], and the observation of the specific band pattern from each primer [25]. Some previous studies used RAPD markers for detection of high levels of genetic polymorphisms by the used radiations [23, 26, 27]. In relation to fast neutron, there are no previous studies that dealt SDS-PAGE and RAPD-PCR for detection of its mutagenic effects and oxidative potential in crop plants. 

In light of the previously mentioned, the main goal of this study is to evaluate and compare the effects of various levels of ROS generated by different irradiation doses of FN and UV-B on PMCs, PGs, and seeds yield of irradiated *Vicia faba* seedling on the cytogenetic and molecular levels for a better understanding of their oxidative stress and mutagenic potential in case of accidental or occupational exposure. 

## 2. Materials and Methods

### 2.1. Plant Material and Germination

Faba bean seeds (*Vicia faba* L. variety Giza 2) were obtained from the Agriculture Research Center in Cairo, Egypt. Seeds were screened for viability and uniformity size and divided into three groups (A, B, and C). Seeds of all groups were sterilized and germinated until seven-day-old seedlings. Seedling of groups (A and B) is irradiated with FN and UV-B (280–320 nm) radiations, respectively, while seedling of group (C) was maintained without irradiation (un-irradiated samples). 

### 2.2. Irradiation Processes

The seedling of group (A) is packed regularly in polyethylene high-density bags and irradiated by fission neutrons from Cf^252^ point source using four fluencies (2.5 × 10^5^, 2.3 × 10^6^, 3 × 10^7^, and 1.5 × 10^8^ n/cm^2^). The source was manufactured by Radiochemical Center Amersham, England, and presented at Biophysics Department, Faculty of science, Zagazig University, Egypt. On the other hand, the seedling of group (B) was exposed to a standard laboratory UV-B (280–320 nm) sterile fluorescent lamp of 500/630 *μ*W/cm^2^ for different time periods (1/2, 1, 2, 3 hours) at a distance of 30 cm from the irradiated seedling. After irradiation processes, groups (A, B, and C) were subdivided and prepared for cytogenetic and molecular analyses.

### 2.3. Cytogenetic Analysis of Meiotic PMCs and PGs

Irradiated and un-irradiated seedlings were transferred immediately to soil and sown in rows under field conditions. A spacing of 30 cm row to row and 15 cm plant to plant were maintained. At maturity, ten flower buds from ten plants for each irradiation dose in addition to un-irradiated ones were collected, fixed immediately in Carnoy's fixative (3 : 1) absolute ethyl alcohol : glacial acetic acid for 24 hours and then stored in refrigerator in 70% ethyl alcohol and finally stained using acido-carmin smear method [28]. Cytogenetic analyses of PMCs selected from six randomly flower buds were scored for 1st and 2nd meiotic anomalies. On the other hand, the pollen fertility test was carried out using the same acido-carmin stain of matured anthers. Pollen grains, which took stain and had a regular outline, were considered as fertile, while empty and unstained ones as sterile [29].

### 2.4. Seed Yield Measurements

Quantitative parameters of six plants irradiated by the various irradiation doses of FN and UV-B radiation mentioned previously were measured as mean number of pods/plant, mean number of seeds/plant, and mean dry weight of 100 seeds compared with those of un-irradiated one. Seeds yielded from irradiated and un-irradiated plants were harvested and analyzed on molecular level using SDS-PAGE analysis of seed storage proteins and RAPD analysis of seed DNA via the polymerase chain reaction (PCR).

### 2.5. SDS-PAGE Analysis of Seed Storage Proteins of *M*_1_ Progeny

Seeds yielded from all irradiated faba beans seedlings by FN and UV-B were used for SDS-PAGE analysis.

#### 2.5.1. Seed Cake and Defatted Meal Preparation

Sterilized seeds were milled and defatted according to [30].

#### 2.5.2. Extraction of Seed Storage Proteins and SDS-PAGE Analysis

The protein extraction technique was employed according to [31]. Sample buffer was added to 0.2 g of seed flour as extraction liquid and mixed thoroughly in an Eppendorf tube by vortex. The extraction buffer contained the following final concentration: 0.5 M Tris-HCl, PH 6.8, 2.5% SDS, 5% urea, and 5% 2-mercaptoethanol. Before centrifugation at 10,000 g for 5 min at 4°C, the sample buffer was boiled for 5 min. SDS-PAGE was performed by a standard method on a vertical slab gel. Bromophenol blue was added to the supernatant as tracking dye to watch the movement of protein in the gel. Proteins profiling of samples was performed using SDS-polyacrylamide gels as described by [32]. Seed proteins were analyzed by SDS-PAGE using 10% polyacrylamide gel. After electrophoresis, the protein bands were visualized by staining with Coomassie brilliant blue G-250. Marker proteins (Fermentas) were used as references. The bands produced in the electropherogram were scored, and their molecular weights were compared to the standard Pharmacia protein marker.

#### 2.5.3. Protein Imaging and Data Analysis

Gel photographing and documentation were carried out using Bio-Rad gel documentation system. The number of bands revealed in each gel lane were counted and compared with each other's using Gel Pro-Analyzer. Quantitative variations in band number as well as band concentration were estimated using BIO-RAD Video densitometer, Model Gel Doc 2000.

### 2.6. RAPD Analysis of Seeds Yield of Irradiated *Vicia faba*

Seeds yielded from irradiated faba beans seedlings by stimulatory FN fluency 2.3 × 10^6^ and UV-B doses for 1 h and inhibitory FN fluency 1.5 × 10^8^ and UV-B doses for 3 h were used for RAPD analysis. 

#### 2.6.1. Isolation of Genomic DNA

Fifty grams of dried seeds of both two doses of FN and UV-B was crushed in a mill and powdered by using a domestic grinder. The powder was sieved using thin mesh and only finely ground powder was kept in refrigerator until DNA extraction. One gram of finely sieved seed powder was taken, and genomic DNA was isolated using Hexadecyl trimethyl ammonium bromide (CTAB) as described by [33].

#### 2.6.2. Quantity and Quality of Isolated DNA

The yield of DNA per gram of seed material extracted was measured by using UV spectrophotometer (Perkin Elmer) at 260 nm and A280 nm. Thepurity of DNA was determined by calculating the ratio of absorbanceat 260/280 nm. For quality and yield assessments, electrophoresis was done of all DNA samples on 0.8% agarose gel, stained with ethidium bromide, and bands were observed in gel documentation system (Alpha Innotech) and compared with the known standard Lambda DNA marker. The gels were visualized and photographed under UV light (Gel documentation system, Bio-Rad).

#### 2.6.3. PCR Amplification Using Random Primers (RAPD)

Briefly, the PCR reaction mixture contains 2.5 *μ*L 10x buffer with 15 mM MgCl_2_ (Fermentas), with 0.25 mM each of dNTP (Sigma), 0.3 *μ*M of the primer, 0.5 unit of Taq DNA polymerase (Sigma), and 50 ng of template DNA. PCR reaction was performed in Palm Cycler (Corbett Research) using the following profile with initial denaturation of 4 min at 95°C followed by 40 cycles of 1 min at 95°C, 1 min at 38°C, and 2 min at 72°C with final extension at 72°C for 10 min and a hold temperature of 4°C at the end. A total of twenty random DNA oligonucleotide primers (10 mer) were independently used in the PCR reactions (UBC, University of British Columbia, Canada) according to [34] with some modifications. Only six primers (A-02, 03, 10, 12, 15, and 17) succeeded to generate reproducible amplified DNA products. The code and sequences of these primers were listed in (Table 4). The amplification products were electrophoresed on 1.5% Agarose gel (Sigma) in TAE buffer (0.04 M Tris-acetate, 1 Mm EDTA, pH 8). The run was performed at 100 V constant voltagesfor one hour. Gels were stained with 0.2 *μ*g/mL ethidium bromide. Bands were detected on UV-transilluminator and photographed by a Polaroid camera.

#### 2.6.4. Data Analysis

Gels were visualized with Photo Print (Vilber Lourmat, France) imaging system, and analysis of RAPD bands was performed by BioOne D++ software (Vilber Lourmat, France). The RAPD bands (markers) were scored as 1 if present and 0 if absent. 

## 3. Results

### 3.1. Cytogenetic Analysis of Meiotic PMCs and PGs

**Figure 1:**
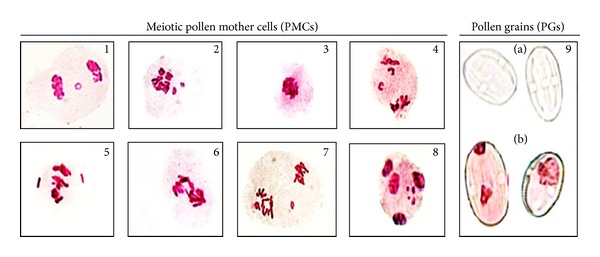
The most common meiotic chromosomal aberrations induced by fast neutron ((1)–(4)) and UV-B radiation ((5)–(8)). (1) Telophase I with micronucleus, (2) disturbed anaphase, (3) sticky metaphase I, (4) sticky anaphase II with two lagging chromosomes, (5) metaphase I with two unoriented chromosomes, (6) sticky anaphase I with bridges, (7) metaphase II with sticky fragment, (8) four micronuclei with sticky telophase II, (9) (a) sterile Pollen grains (colorless), and (b) fertile one (color).

The ability of fast neutron and UV-B radiation to exert genotoxic action on PMCs DNA in 1st and 2nd meiotic divisions was observed in spite of long period of recovery (Table 1) and (Figure 1, (1)–(8)). Both radiations induced a wide range of meiotic abnormalities extended in 1st and 2nd meiotic divisions after all irradiation doses are compared to un-irradiated samples. Moreover, the types and frequencies of anomalies in meiotic PMCs were linearly linked to the irradiation exposure doses. The maximum values of meiotic-PMCs abnormalities were 65.91 ± 1.00% at FN fluency (1.5 × 10^8^ n/cm^2^) and 55.13 ± 0.18% at UV-B dose after 3 hours. The most frequent types of PMCs abnormalities induced by various fluencies of FN were stickiness, chromosomal disturbances, unoriented chromosomes, and chromosomal fragmentation, whereas the anomalies induced by irradiation doses of UV-B were meiotic micronuclei, chromosomal fragmentation, stickiness, and bridges (Figure 1). On the other hands, the pollen grains fertility was dose dependent as evident from its reduction by all irradiation doses of FN and UV-B expect FN fluency (2.3 × 10^6^) and UV-B dose for 1 h compared to un-irradiated one (Table 1) and (Figure 1  (a) and (b)). The maximum reduction of PGs fertility was observed at FN fluency (2.3 × 10^8^) and UV-B dose for 3 h which reached the values of (35.88 ± 0.12% and 25.54 ± 0.26%), respectively; this indicated that the irradiation exposure doses of UV-B were more effectiveness in reduction of PGs fertility than irradiation fluencies of FN. On the other hand, FN fluency (2.3 × 10^6^) and UV-B dose for 1 h showed improvement in the values of PGs fertility reaching (96.95 ± 0.12% and 93.72 ± 0.25%), respectively, nearly similar to the value of un-irradiated sample which reached (99.98 ± 0.12%).

### 3.2. Parameters of Seed Yield Productivity

The different parameters of seeds yield productivity of irradiated faba beans seedlings that were represented in the mean number of pods/plant, number of seeds/pod, and average weight of 100 seeds/gm were dose-dependent as evident from their reduction by most irradiation doses of FN and UV-B radiations expect FN fluency (2.3 × 10^6^) and UV-B dose after 1 h that showed improvement in parameters of seeds yield productivity (Table 2). This indicated that these doses may be having bio-positive or stimulation effects that can be useful in induction of mutations in faba beans plants.

### 3.3. SDS-PAGE Analysis

**Figure 2:**
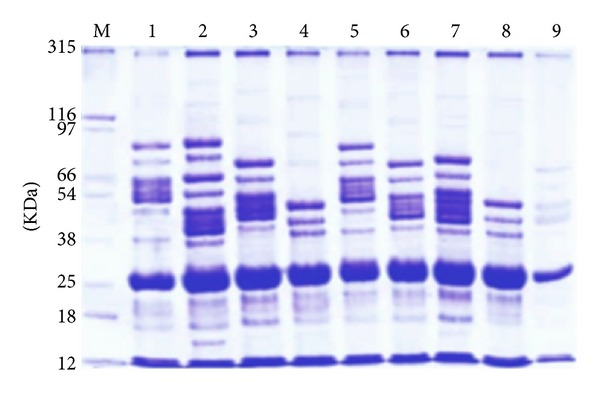
SDS-PAGE banding patterns of storage protein in seeds yielded from irradiated *Vicia faba* seedlings by various doses of fast neutron and UV-B. M: Protein marker, (1) (2.5 × 10^5^ n/cm^2^), (2) (2.3 × 10^6^ n/cm^2^), (3) (3 × 10^7^ n/cm^2^), (4) (1.5 × 10^8^ n/cm^2^), (5) un-irradiated seeds, (6) UV-B dose for 1/2 hour, (7) UV-B dose for 1 hour, (8) UV-B dose for 2 hours, and (9) UV-B dose for 3 hours.

Each irradiation dose of FN and UV-B used in the current study exhibited distinctive quantitative and qualitative alterations in electrophoretic banding pattern of total seed proteins yielded from irradiated faba bean seedlings compared to un-irradiated ones. These protein alterations are based on changes in bands molecular weights (MWs), bands intensities, fractionation of some bands, appearance of new bands (unique bands), and disappearance of some bands (polymorphic bands) as shown in (Table 3) and (Figure 2). SDS-PAGE analysis revealed total of (111) polypeptides bands with different MWs that ranged from 315 to 12 KDa. Out of which, 22, 3, and 3 bands were polymorphic, unique, and monomorphic bands, respectively. Two unique bands with MWs (175 and 29 KDa) were recorded at FN fluency (2.3 × 10^6^), while one unique band with MW (120 KDa) was observed only at UV-B dose for 1 h. These unique bands can be used as markers for these irradiation doses. The total value of polymorphism revealed by SDS-PAGE was (88%). On the other hands, the maximum number of polypeptide bands was (18 bands) with value (16.22%) observed at FN fluency (2.3 × 10^6^), whereas the minimum number of bands was (7 bands) with value (6.31%) observed at UV-B dose for 3 h compared with number of polypeptides bands in un-irradiated sample which reached (14 bands) with value (12.61%). 

### 3.4. RAPD-PCR Analysis

**Figure 3:**
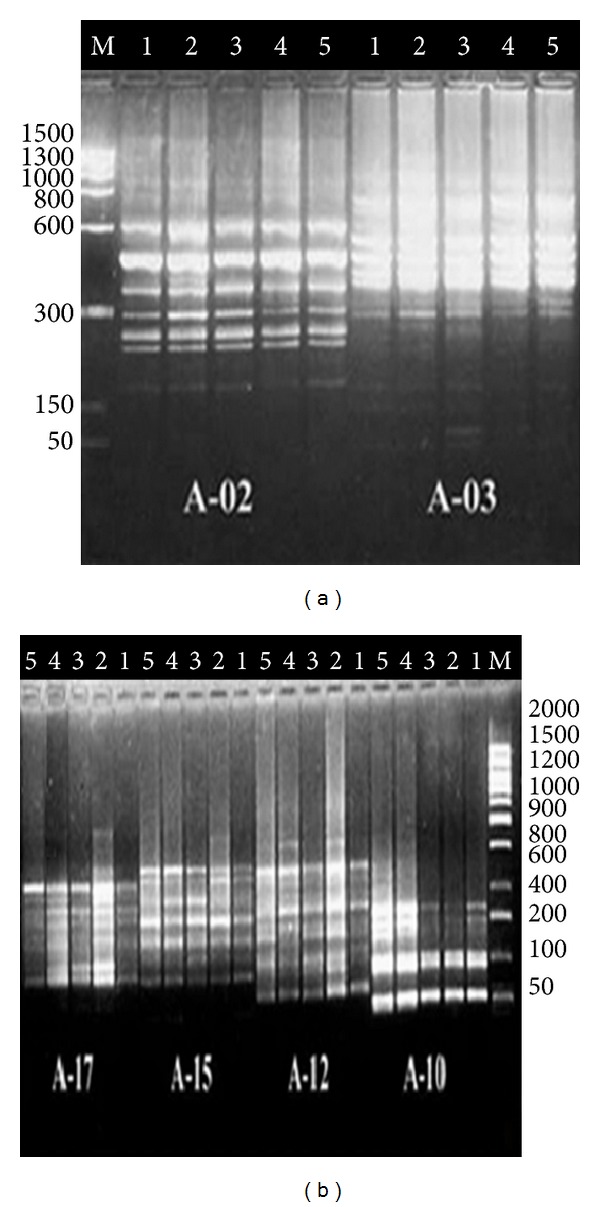
(a) and (b) DNA banding pattern of RAPD analysis of seeds yielded from irradiated *Vicia faba* seedlings generated by the six primers; (1) (1.5 × 10^8^ n/cm²), (5) UV-B dose for 3 h as bio-negative irradiation doses, (2) (2.5 × 10^6^ n/cm²), (4) UV-B dose for 1 h as bio-positive irradiation doses of each FN and UV-B, respectively, and (3) un-irradiated sample. M: Lambda DNA marker.

RAPD analysis was employed in the present study to evaluate the extent of the DNA alterations in seeds yielded from irradiated faba beans seedlings by the two bio-positive doses (FN fluency (2.3 × 10^6^) and UV-B dose for 1 h) and the two negative doses (FN fluency (2.3 × 10^8^) and UV-B dose for 3 h). Twenty random primers were used for the RAPD analysis, in which only six primers of them (A-02, 03, 10, 12, 15, and 17) succeeded to produce clear reproducible DNA bands and gave satisfactory results with many alterations in the RAPD profiles as shown in (Table 4) and (Figure 3). In total, two hundred forty-eight (248) reproducible DNA bands were scored after using the six primers (with an average of 41 bands/primer). Out of which 76 bands were polymorphic with value (30.65%), 27 bands were unique with value (10.89%), and 8 bands were monomorphic with value (3.23%). Moreover, the total value of polymorphism generated by six primers reached the value of (90.80%). The maximum number of gene products (60 bands) with value (24.19%) was observed at FN fluency (2.3 × 10^6^), whereas the minimum number of bands (45) with value (18.15%) was at UV-B dose for 3 h. On the other hand, the highest number of gene products (60 bands) was generated by primer A-03, whereas the lowest number (31 bands) was generated by the primer A-17. Furthermore, the highest value of polymorphism (100%) was revealed by the primer A-03, whereas the lowest one (58.82%) is revealed by the primer A-02.

## 4. Discussion

### 4.1. Cytogenetic Analysis of Meiotic PMCs, PGs, and Seed Yield Productivity

Cytogenetic test is considered to be indicator of oxidative potential, genotoxicity, and estimation of the mutagen potency in meiotic PMCs and PGs [12, 19]. The present investigation observed that all irradiation exposure doses of FN and UV-B exhibited special interference with meiotic PMC and PGs leading to genotoxic effects except two doses (FN fluency (2.3 × 10^6^) and UV-B dose for 1 h) that showed improvement in meiotic PMC, pollen fertility, and consequently, parameters of seed yield productivity. This improvement may be due to the antioxidant defense system of *Vicia faba* plant which allows the toxic-free radical oxygen intermediates to perform useful biological functions without too much damage [5, 7]. Therefore, these irradiation doses may be having bio-positive or stimulation effects that may lead to inducing useful mutations as a new source of altered *Vicia faba* germplasm. In this respect, some previous studies indicated that the FN and UV-B irradiations had bio-positive effects at specific dose [35, 36], respectively. On the contrary, the remained irradiation doses of FN and UV-B induced genotoxic and oxidative action in meiotic-PMC and PGs of faba beans. These actions may be due to the induction of oxidative damage in these cells by production of the free radical oxygen that lead to higher frequency of chromosomal aberrations and DNA damage which in turn can affect the vigor, pollen grains fertility and likely to persist in seeds yield or even longer due to the accumulative genotoxicity and chromosomal aberrations [37]. Moreover, pollen grains which have no cytoplasm content and fail to pick up the acido-carmin stain were sterile [29]. Each of FN and UV-B radiations can induce ROS in PMCs by special interference with DNA leading to induction of structural changes in DNA, such as chromosomal rearrangement, strand breaks, base deletions, pryrimidine dimers, cross-links and base modifications, mutations, and other genotoxic effects [5, 6]. These DNA damages may influence the expression of a number of genes leading to alteration in proteins that control many metabolic processes like plant development, cell cycle, fertilization, and seed formation [2]. 

### 4.2. SDS-PAGE Analysis of Seed Storage Proteins of *M*_1_ Progeny

The present study observed that SDS-PAGE analysis exhibited distinctive qualitative and quantitative alterations in electrophoretic SDS-proteins stored in seeds yielded from irradiated faba beans seedlings. These alterations are based on variations in molecular weights and intensities of polypeptides bands as well as gain or loss of protein bands that led to highly levels of protein polymorphism. Electrophoretic analysis of protein provides information concerning the structural genes and their regulatory systems that control the biosynthetic pathways of that protein. Each polypeptide band represents the final products of a transcriptional and translational events occurring due to active structural genes [38]. The changed protein products caused by dependent-irradiation exposure doses may result from base changes in DNA or altering protein sites or changes in amino acid sequences or frame shift mutations. Additionally, they may serve as genetic markers because they can be quite polymorphic and their variability is generally highly heritable [39]. 

The appearance of new bands (unique bands) may be explained on the basis of mutational events at the regulatory system of unexpected gene(s) or on the basis of band subfractionation which could be attributed to the cytological anomalies in PMCs leading to gene duplication followed by the occurrence of point mutation that encoded the fractionated band [40] or result from different DNA structural changes (breaks, transpositions, deletion, etc.) which led to change in amino acids and consequently protein formed [39]. On the other hand, the disappearance of some protein bands which led to formation of polymorphic bands could be attributed to the loss of genetic material which may be due to the cytogenetic anomalies in PMCs such as chromosomal laggards, free, fragmentations, bridges, micro- and multinucleate, or the breaking of a small number of peptide bonds to form polypeptides of shorter length than the original protein [38]. Furthermore, the changes in band intensity could be interpreted on the basis of gene duplication or point mutation that leads to production of shorter and longer polypeptide chains and alteration in the structural genes which may be due to the changes in regulator gene(s) expression [41]. The distinction protein polymorphisms shown between irradiated samples in the present study may be resulted from insertions or deletions between mutated sites of protein bands and could be used as biomarkers for identification of irradiated plants [39]. Additionally, high radionuclide content of plants causes alterations in the relative mobility of bands, intensities, expression of new proteins, and suppression of some proteins.

The result obtained in this study indicated that the UV-B doses for (2 and 3 h) may cause highly oxidative protein cross-links due to alteration of the expression of several genes that leading to proteins denaturation and disappearance of numerous bands due to aggregation or cross linking of individual polypeptide chains [42] or alteration of the expression of several genes through nonspecific signaling pathways [5]. On the other hand, the neutron fluency (2.3 × 10^6^ n/cm^2^) could show bio-positive (stimulation) effects by appearance of numerous new bands which can be valuable in the fields of genetic as mutant lines.

### 4.3. RAPD-PCR Analysis of DNA of Seed Yield of *M*_1_ Progeny

RAPD assay used in the current study showed that various irradiation doses of the FN and UV-B exhibited distinctive qualitative and quantitative alterations in the RAPD profiles based on gene products, the amplified DNA sizes, their intensities, and appearance or disappearance of DNA bands that led to generation highly levels of DNA polymorphism. Variations in the characteristic DNA banding pattern generated by RAPD analysis may be caused by rearrangements of the genomic DNA, base pair deletions, mutations, inversions, translocations, and transpositions within base pair sequences of DNA which result in the loss or gain of DNA bands resulting in different DNA lengths and consequently highly level polymorphisms [43]. In this regard, [44] revealed that highly level of DNA polymorphisms generated by RAPD are the reflection of structural changes in the genomic DNA that alter the distance between two annealing sites and delete an existing site of new one or reflection of variation in gene expression which would be a better parameter to measure the pattern of genetic variations. They also concluded that deletion or insertion of the amplified regions or changes of nitrogenous base that alter primer binding sites will result polymorphisms in RAPD profile. On the other hand, appearance of new DNA bands is usually resulting from different DNA structural changes (breaks, transpositions, deletion, etc.), while disappearance of some bands and band intensity may correlate with the level of photoproducts in DNA template after irradiation which can reduce the number of binding sites for Taq polymerase and the starting copy number of a particular DNA sequence within genome [26]. Furthermore, the disappeared DNA bands in some irradiated samples may be due to deletion of DNA segments that is the predominant radiation-oxidative damage in irradiated plant cells. This DNA deletion may be caused by (1) misrepair of two separate double-strand breaks in a DNA molecule with joining of the two outer ends and loss of the fragment between the breaks or (2) the process of cleaning (enzyme digestion of nucleotides, the component molecules of DNA) the broken ends before rejoining to repair one double-strand break [44]. 

The current study investigated that DNA alterations in RAPD profile could be explained on the basis of the biological way by which the radiation type interacts with DNA, by producing their own ROS through the direct and/or indirect effect in the irradiated cells. In relation to ROS generated in faba bean seedlings by bio-negative fluencies of FN can induce DNA deletions in nucleus and chromosome that range in size from a few base pairs to several megabases [14]. These DNA deletions can lead to increasing of the level of DNA break formation by producing different intragenic mutations with respect to the size of deletions that reflect differences in the nature of the DNA damage by generating oxygen species such as hydroxyl (OH) in aqueous media [14, 26]. Whereas ROS generated by bio-negative doses of UV-B radiation is capable of inducing several major types of DNA lesions such as DNA strand breaks, deletion or insertion of base pairs, pyrimidine dimers, cross-links, and base modification, such as alkylation and oxidation. Additionally, these DNA breaks can result from DNA damage by free radicals or from DNA replication, repair, transcription processes, and chromatin condensation and decondensation [2]. The breaks and perturbation of the molecular structures of deoxyribonucleic acids are manifested as chromosomal aberrations in meiotic-PMCs. UV-B can influence metabolic processes of plants either through direct damage including DNA damage and protein denaturation, which often cause heritable mutations affecting various metabolic processes or via various regulatory effects. These effects could adversely affect plant growth, development, and morphology, especially the productivity of sensitive crop species [45].

## 5. Conclusions

The present study observed that the cytogenetic analysis of meiotic PMCs and PGs in addition to SDS-PAGE and RAPD analyses of proteins and DNA of seeds yielded from irradiated faba beans seedlings by neutron fluency (2.3 × 10^6^) and UV-B dose for 1 h was recorded as stimulatory effects for PMCs, PGs viability and parameters of seed productivity. These data are supported and confirmed by generation of numerous protein bands and high number of gene products generated from appearance of new protein and DNA bands, respectively, at these doses. Moreover, neutron fluency (2.3 × 10^6^) was more stimulatory and effective as compared with UV-B dose for 1 h which in turn was higher than un-irradiated ones. In view of this, the current study proved that cytogenetic analysis alone cannot reveal alterations at the genome level; therefore it must be augmented with gene expression analyses represented in alteration in electrophoretic SDS-protein profiles using SDS-PAGE and DNA levels, changes in RAPD profiles using RAPD-PCR techniques that offered a useful molecular marker for evaluation of oxidative stress and mutagenic effects of various levels of ROS induced by irradiation exposure doses of FN and UV-B in faba beans cells. Finally, the current study proved that neutron fluency (2.3 × 10^6^) and UV-B dose for 1 h were recorded as bio-positive or stimulation effects. Therefore, they may lead to induction of useful mutation in crop plants and can be useful as a new source of altered germplasm which may be valuable in the field of genetic and crop improvements.

## Figures and Tables

**Table 1:** Number, frequencies of abnormal PMCs in 1st and 2nd meiotic divisions, frequencies of meiotic abnormalities, types of abnormal meiotic PMCs, and percentages of fertile PGs after irradiation of *Vicia faba* seedlings by various irradiation doses of FN and UV-B.

Irradiation types	Doses	1st meiotic division		2nd meiotic Division		Total no. of PMCs	Total no. of abnormal PMCs	% of abnormal PMCs ± SE	Types and frequency of meiotic abnormalities of PMCs								PGs	
		Total no. of PMCs	% of abnormal Cells	Total no. of PMCs	% of abnormal Cells				Stickiness	Disturbed	Unorient.	Micronuclei + fragments	Lagging + free	Bridge	Tripolar	Multipolar	No. of PGs	Mean % of fertility
Un- irradiated	0.00	835	0.00	1050	0.00	1885	0	0	0.00	0.00	0.00	0.00	0.00	0.00	0.00	0.00	600	99.98 ± 0.12

FN fluencies (Φ) n/cm²	2.5 × 10^5^	541	38.26	339	47.79	880	369	38.80 ± 0.54	19.56	30.37	14.43	13.90	8.52	2.64	9.13	1.60	600	88.90 ± 1.40
	2.3 × 10^6^	446	15.47	505	14.85	951	145	14.23 ± 0.95	25.06	37.95	12.51	5.82	6.71	6.26	3.58	1.12	600	99.96 ± 0.12
	3.0 × 10^7^	515	58.64	440	42.50	955	498	52.15 ± 2.17	25.56	27.37	10.30	13.73	4.61	8.40	7.59	2.44	600	65.10 ± 0.06
	1.5 × 10^8^	412	66.50	336	65.18	748	493	65.91 ± 1.00	41.92	12.47	11.86	15.75	6.95	3.48	4.94	2.69	600	35.88 ± 0.12

UV-B doses *μ*w/cm²	1/2 h	526	33.08	435	38.39	955	341	35.71 ± 0.79	12.00	7.84	20.85	42.22	3.82	10.04	0.88	2.35	600	81.99 ± 0.02
	1 h	459	21.70	435	19.06	1158	236	20.38 ± 0.54	22.40	5.91	8.93	33.20	4.48	19.55	3.26	3.26	600	96.72 ± 0.25
	2 h	424	49.06	450	43.55	874	404	46.22 ± 1.12	31.68	4.70	8.17	25.57	6.44	20.96	1.49	1.00	600	57.77 ± 0.23
	3 h	680	49.71	421	49.64	1101	607	55.13 ± 0.18	37.50	8.25	10.51	31.16	3.44	6.34	1.27	1.74	600	25.54 ± 0.26

**Table 2:** The productivity parameters of seeds yielded from irradiated *Vicia faba* seedlings by various irradiation doses of FN and UV-B.

Irradiation types	Doses	Seed yield parameters		
		No. of pods/plant	No. of seeds/plant	Average wt. of 100 seeds/gm
FN fluencies (Φ) n/cm²	0.00	21 ± 1.90	62 ± 2.23	72.80 ± 1.30
	2.5 × 10^5^	14 ± 2.10	53 ± 1.20	65.60 ± 1.17
	2.3 × 10^6^	28 ± 1.2	70 ± 2.00	80.60 ± 2.30
	3.0 × 10^7^	12 ± 3.10	33 ± 3.21	56.20 ± 3.00
	1.5 × 10^8^	10 ± 1.50	19 ± 1.60	40.20 ± 1.72

UV-B doses *μ*w/cm²	0.00	21 ± 1.00	62 ± 2.23	72.80 ± 1.30
	1/2 h	15 ± 1.7	45 ± 1.2	56.00 ± 2.33
	1 h	23 ± 2.50	68 ± 2.30	75.43 ± 1.76
	2 h	12 ± 1.5	27 ± 1.50	44.12 ± 2.00
	3 h	8 ± 1.10	11 ± 1.76	28.45 ± 1.66

**Table 3:** Electrophoretic banding analysis of seeds storage proteins yielded from irradiated *Vicia faba* plant by various doses of FN and UV-B.

No.	MW/KDa	Protein marker		Fluencies of FN radiation (n/cm²)								Un-irritated sample		Various doses of UV-B radiation (*μ*w/cm²)									Polymorphism types
				2.5 × 10^5^		2.3 × 10^6^		3.0 × 10^7^		1.5 × 10^8^				1/2 h			1 h		2 h		3 h	
		Lane M		Lane 1		Lane 2		Lane 3		Lane 4		Lane 5		Lane 6			Lane 7		Lane 8		Lane 9	
		KDa	%	KDa	%	KDa	%	KDa	%	KDa	%	KDa	%	KDa		%	KDa	%	KDa	%	KDa	%
1	315	+	+	+	32.77	+	106.58	+	106.84	+	66.82	+	87.95	+		90.73	+	101.3	+	96.49	+	52.91	M
2	181	−	−			−	−	−	−	−	−	+	32.69	−		−	−	−	−	−	−	−	P
3	175	−	−	−	−	+	17.83	−	−	−	−	−	−	−		−	−	−	−	−	−	−	U
4	158	−	−	−	−	−	−	+	16.35	−	−	−	−	+		11.28	+	17.83	+	12.57	−	−	P
5	145	−	−	−	−	−	−	−	−	+	14.3	−	−	−		−	+	10.37	−	−	−	−	P
6	128	−	−	−	−	+	17.83	−	−	−	−	+	15.39	−		−	−	−	−	−	−	−	P
7	120	−	−	−	−	−	−	−	−	−	−	−	−	−		−	+	9.43	−	−	−	−	U
8	116	116	8.86	−	−	+	10.54	+	15.16	−	−	−	−	+		11.28	+	12.48	−	−	−	−	P
9	97	97	29.84	−	−	−	−	−	−	+	9.23	−	−	−		−	−	−	+	9.43	−	−	P
10	86	−	−			+	106.00	+	125.86	+	5.34	+	99.46	−		−	−	−	−	−	−	−	P
11	75	−	−	+	81.51	+	103.47	−	−	−	−	+	99.23	+		114.7	+	118.96	−	−	+	9.22	P
12	66	66	33.00	+	77.69	+	118.97	+	106.00	−	−	+	105.23	+		111.6	+	118.96	−	−	+	−	P
13	60	−	−	−	−	−	−	−	−	−	−	+	128.02	−		−	−	−	−	−	−	7.22	p
14	54	−	−	+	77.69	+	95.21	−	−	−	−	+	126.51	−		−	+	−	−	−	−	−	P
15	50	−	−	+	91.41	+	94.02	+	86.79	−	−	+	117.37	−		−	+	126.51	−	−	−	−	P
16	45	−	−	+	52.69	+	101.95	+	125.54	+	100.21	+	138.29	+		109.63	+	77.41	+	58.55	+	14.84	M
17	40	−	−	+	14.84	−	−	+	38.65	+	73.35	+	56.23	+		55.71	+	77.19	−	−	+	28.59	P
18	38	38	32.88	+	21.36	+	14.57	−	−	−	−	−	−	−		−	−	−	−	−	−	−	P
19	29	−	−	−	−	+	11.62	−	−	−	−	−	−	−		−	−	−	−	−	−	−	U
20	25	−	−	+	225.17	+	232.77	+	266.37	+	256.43	+	267.16	+		261.93	+	237.79	+	231.78	+	−	p
21	23	−	−	−	−	+	19.42	+	41.76	+	77.98	−	−	+		78.44	+	35.64	+	36.11	−	118.23	P
22	18	18	43.44	+	41.76	+	56.82	+	94.7	+	44.88	−	−	+		61.21	−	−	+	29.95	−	−	P
23	16	−	−	+	16.23	+	26.58	+	17.85	+	17.34	+	9.28	+		12.48	+	10.46	+	29.95	−	−	P
24	14	−	−	−	−	+	26.58	−	−	−	−	−	−	−		−	+	11.07	−	−	−	−	P
25	12	12	103.15	+	134.29	+	173.34	+	188.58	+	174.19	+	177.85	+		179.34	+	137.12	+	146.44	+	128.09	M

Total bands of each lane				12		18		13		11		14		12			15		9		7		
% Total bands				10.81		16.22		11.71		9.91		12.61		10.81			13.51		8.11		6.31		

				Polymorphic bands				Unique bands				Monomorphic bands					% of polymorphism						
Total protein bands of all lanes = 111				No. of bands		%		No. of bands		%		No. of bands			%								
																	88						
				22		19.82		3		2.70		3			2.70								

**Table 4:** RAPD-PCR amplification products of DNA extracted from seeds yielded from irradiated *Vicia faba *by FN and UV-B using six random primers.

			Total no. of scorable bands							Polymorphic bands							
Primers	Sequence of primers from 5′→3′	Amplicon lengths (pb)	Fluencies of fast neutrons radiation (n/cm²)		Control	Various doses of UV-B radiation (*μ*w/cm²)		Total no.	% of total bands	No. of unique bands	% of unique bands	No. of nonunique bands	% of nonunique bands	% of polymorphic bands	No. of monomorphic bands	% of monomorphic	% of Total polymorphism
			1.5 × 10^8^	2.3 × 10^6^		1 h	3 h										
			Lane 1	Lane 2	Lane 3	Lane 4	Lane 5										
A-02	TGCCGAGCTG	1500–50	12	13	10	10	12	57	22.98	4	7.02	9	15.79	17.54	4	7.02	58.82
A-03	AGTCAGCCAC	1300–50	11	15	13	11	10	61	24.60	6	9.83	15	24.59	34.43			100.00
A-10	GTGATCGCAG	550–50	6	5	4	9	6	30	12.10	3	10.00	4	13.33	20.00	1	3.33	75.00
A-12	TCGGCGATAG	900–65	5	8	7	7	9	35	14.11	4	11.43	7	20.00	31.43	1	2.86	91.67
A-15	TTCCGAACCC	700–75	8	8	7	6	5	34	13.71	5	14.71	5	14.71	23.53	1	2.94	72.73
A-17	GACCGCTTGT	800–65	5	11	5	7	3	31	12.50	5	16.13	9	29.03	45.16	1	3.23	93.33

	Overall total DNA bands %Total DNA bands		47 18.95	60 24.19	46 18.55	50 20.16	45 18.15	248		27	10.89	49	19.78	30.65	8	3.23	90.80
